# The ability of the rumen ciliate protozoan *Diploplastron affine* to digest and ferment starch

**DOI:** 10.1007/s12223-012-0146-1

**Published:** 2012-04-19

**Authors:** K. Wereszka, T. Michałowski

**Affiliations:** Department of Ruminant Physiology of Nutrition, The Kielanowski Institute of Animal Physiology and Nutrition, Polish Academy of Sciences, Instytucka 3, 05-110 Jablonna near Warsaw, Poland

## Abstract

The ciliate *Diploplastron affine* is known as a common species of the rumen fauna in cattle and sheep. This protozoon is able to digest cellulose, whereas its amylolytic activity is not well known. The objective of the reported studies was to examine the ability of *D. affine* to digest starch and to use this polysaccharide to cover the requirement for energy. The enzymatic studies showed that the protozoal cell extract degraded starch to reducing products with the rate being equivalent to 2.4 ± 0.47 μmol/L glucose per mg protein per min. Maltose, maltotriose and a small quantity of glucose were the end products of starch degradation. The degradation rate of maltose was only 0.05 μmol/L glucose per mg protein per min. Two peaks in α-amylase and a single peak in maltase activity were found following molecular filtration of ciliate cell extract, whereas three starch-degrading enzymes were identified by a zymographic technique. Incubation of the bacteria-free ciliates with starch in the presence of antibiotics resulted in a release of volatile fatty acids with the net rate of 25 pmol per protozoan per h. Acetic acid followed by butyric acid was the main product of starch fermentation. The results confirmed the ability of *D. affine* to utilize starch in energy-yielding processes.

## Introduction

The ciliate *Diploplastron affine* is a common species of rumen fauna in the domesticated ruminants (Dogiel 1927). To date, the fibrolytic properties of this protozoan were studied by Coleman (1985) whereas its ability to digest and utilize starch is not well known. The objectives of the reported studies were to examine the ability of *D. affine* to digest starch and to use this polysaccharide to cover the requirement for energy.

## Material and methods

The ciliates were isolated from the rumen fluid of sheep. The cell extract for enzymatic studies was obtained by homogenization of purified protozoa and removal of the particulate matter by centrifugation. To restrict the bacteria, the ciliates were incubated overnight with ampicillin, streptomycin and chloramphenicol prior to the homogenization. The amylolytic activity of the ciliates was examined by quantification of products released during incubation of the cell extract with appropriate substrates. Fractionation of the crude enzyme preparation was performed by molecular filtration using a glass column packed with Sephadex G-150. Enzymes were identified by a zymography technique following separation of protein on polyacrylamide gel (Gabriel and Wang 1969). Volatile fatty acids (VFA) were measured chromatographically during the incubation of the protozoa with starch and antibiotics.

## Results and discussion

**Fig. 1:**
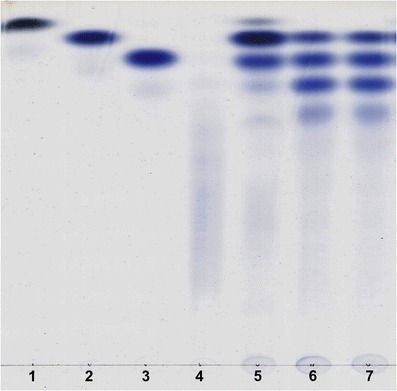
Thin layer chromatography of the products of starch degradation by crude enzyme preparation and its two fractions. *Lanes 1–4* standards of glucose, maltose, maltotriose and maltooligosaccharides, respectively; *lanes 5–7* products of starch degradation by ciliate cell extract and its fractions 21 and 30 (see also Fig. 2)

**Fig. 2:**
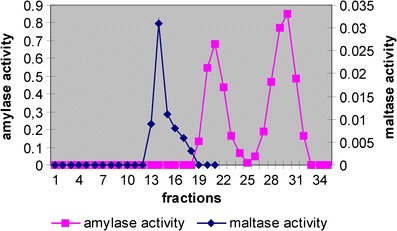
Distribution of amylase and maltase activity over the fractions of crude enzyme preparation separated by molecular filtration method on Sephadex G-150

**Fig. 3:**
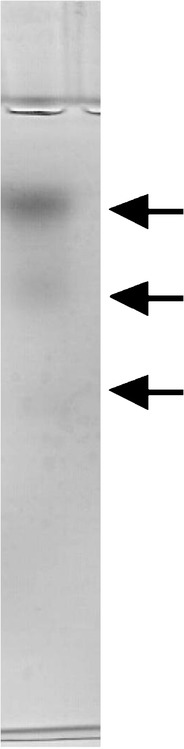
Amylolytic enzymes (1, 2, 3) identified by zymogram technique in combination with non-denaturating polyacrylamide gel electrophoresis of cell extract protein of *D. affine* protein. The activities were visualized by staining with 2,3,5-triphenyltetrazolium chloride

Ciliate *D. affine* possesses enzymes degrading starch and its derivatives. Protozoal cell extract prepared from ciliates incubated with antibiotics released reducing sugars from starch, dextrin, maltose and isomaltose (Fig. 1, Table 1). On the other hand, pullulanase activity was not found (Table 1). Similar results were obtained earlier when amylolytic activity of *Eudiplodinium maggii* was studied (Bełżecki et al. 2007). The ciliate *D. affine* ingested and fermented starch, releasing about 45 pmol VFA per protozoan per h. The control cultures produced only about 20 pmol VFA per protozoan per h (Table 2). This indicates that the products of starch degradation were utilized to cover the requirement of ciliates for energy. The determination of amylase activity after the separation of protozoal protein by molecular filtration revealed that this activity formed two distinct peaks (Fig. 2). The single peak of maltase activity was also present there. Conversely, Bełżecki et al. (2007) found only a single peak of amylolytic activity when the crude enzyme preparation of *E. maggii* was fractionated by ion exchange chromatography. On the other hand, three starch-degrading enzymes were identified by a zymographic technique following the separation of *D. affine* protein by native polyacrylamide gel electrophoresis (Fig. 3). For comparison, four such enzymes were found in *E. maggii* crude enzyme preparation (Bełżecki et al. 2007). Ciliate *D. affine* belongs to the rumen microorganisms which are able to digest starch and to utilize the obtained products as a carbon source in energy-yielding processes.

**Table 1:** The digestion characteristics of starch, dextrin, maltose and isomaltose by cell extract of *D. affine*

Degraded substrate^a^	Degradation rate	Optimum pH	Optimum temperature
Starch	2.42 ± 0.47	5.5	50
Dextrin	1.25 ± 0.25	5.5	45
Maltose	0.05 ± 0.01	7.0	40
Isomaltose	0.025 ± 0.01	5.5	50

Digestion characteristics: rate (in millimoles per litre of released glucose per milligram protein per minute), optimum pH and temperature (in degrees Celsius) of hydrolysis of particular substrates; means ± SD of triplicates

^a^Pullulan was not detected

**Table 2:** The production rate of VFA (in picomoles per protozoon per hour) and proportion of particular acids in a total of VFA (moles/mole)

Acid	Ciliate^a^	Ciliate + starch^a^
Acetic	70.5 ± 0.37	67.9 ± 0.49
Propionic	10.2 ± 0.59	10.3 ± 0.34
Butyric	19.2 ± 0.81	21.8 ± 0.83
Total VFA	19.9 ± 3.28	44.9 ± 11.46

^a^All values are means ± SD of triplicates
